# The Vicious Cycle of Type 2 Diabetes Mellitus and Skeletal Muscle Atrophy: Clinical, Biochemical, and Nutritional Bases

**DOI:** 10.3390/nu16010172

**Published:** 2024-01-04

**Authors:** Jose M. Lopez-Pedrosa, Maria Camprubi-Robles, German Guzman-Rolo, Andres Lopez-Gonzalez, Jose Manuel Garcia-Almeida, Alejandro Sanz-Paris, Ricardo Rueda

**Affiliations:** 1 Abbott Nutrition R&D, 18004 Granada, Spain; maria.camprubirobles@abbott.com (M.C.-R.); andres.lopez1@abbott.com (A.L.-G.); ricardo.rueda@abbott.com (R.R.); 2 Abbott Nutrition R&D, 28050 Madrid, Spain; german.guzman1@abbott.com; 3 Department of Endocrinology and Nutrition, Virgen de la Victoria Hospital (IBIMA), Malaga University, 29010 Malaga, Spain; jgarciaalmeida@gmail.com; 4 Nutrition Unit, Universitary Hospital Miguel Servet, Isabel the Catholic 1-3, 50009 Zaragoza, Spain; asanzp@salud.aragon.es

**Keywords:** diabetes mellitus, malnutrition, muscle mass, muscle strength, obesity, sarcopenia, skeletal muscle atrophy

## Abstract

Today, type 2 diabetes mellitus (T2DM) and skeletal muscle atrophy (SMA) have become increasingly common occurrences. Whether the onset of T2DM increases the risk of SMA or vice versa has long been under investigation. Both conditions are associated with negative changes in skeletal muscle health, which can, in turn, lead to impaired physical function, a lowered quality of life, and an increased risk of mortality. Poor nutrition can exacerbate both T2DM and SMA. T2DM and SMA are linked by a vicious cycle of events that reinforce and worsen each other. Muscle insulin resistance appears to be the pathophysiological link between T2DM and SMA. To explore this association, our review (i) compiles evidence on the clinical association between T2DM and SMA, (ii) reviews mechanisms underlying biochemical changes in the muscles of people with or at risk of T2DM and SMA, and (iii) examines how nutritional therapy and increased physical activity as muscle-targeted treatments benefit this population. Based on the evidence, we conclude that effective treatment of patients with T2DM-SMA depends on the restoration and maintenance of muscle mass. We thus propose that regular intake of key functional nutrients, along with guidance for physical activity, can help maintain euglycemia and improve muscle status in all patients with T2DM and SMA.

## 1. Introduction

For more than a decade, researchers have recognized an overlap between the metabolic conditions of skeletal muscle atrophy (SMA) and type 2 diabetes mellitus (T2DM) [1,2,3]. This concurrence is observed in adults of all ages, but it is increasingly common in adults of older age [1,2,4,5,6]. SMA is defined as the wasting or loss of muscle tissue; SMA results when there is an imbalance between the synthesis and degradation of muscle proteins [7]. T2DM is a prevalent metabolic disorder characterized by an alteration in carbohydrate metabolism resulting from decreased insulin sensitivity in target tissues along with a relative deficiency of insulin secretion. T2DM is also characterized by abnormalities in the metabolism of proteins and lipids [8,9].

A wide range of causes contribute to the overlap of T2DM-SMA and associated adverse consequences. These include malnutrition (over- and under-nutrition, especially when caused by the presence of other chronic and acute health conditions) and low physical activity. Notably, chronic health conditions like obesity and T2DM are associated with inflammation and oxidative stress, in turn leading to unbalanced protein turnover, loss of muscle tissue, and weakness [10,11,12]. Such muscle loss can be further worsened by the intramuscular deposition of fat, leading to poor muscle function [13]. SMA in overweight or obese adults, called sarcopenic obesity, is common but may not be recognized due to the excess body weight [14,15]. In other cases, acute critical illnesses, cancer, and other chronic inflammatory diseases can lead to undernutrition and loss of body weight, in turn hastening a decline in muscle mass and strength [10,16]. In addition, sedentary behavior—due to illness, disability, or lifestyle—further increases the risk of muscle loss [17]. Such associations between skeletal muscle loss, malnutrition, low physical activity, and T2DM are common in adults ≥ 65 years, but the relationship can begin even before age 65 years and worsen over time [18,19,20].

Older age itself represents a prominent risk factor for SMA because of its age-related vulnerability to diseases that impair nutrient intake and use and low physical activity due to overweight/obesity, disability, and immobility [21]. Further, older people commonly experience anabolic resistance of muscle, which makes muscle restoration more difficult [21]. Also, muscle loss with aging is common; muscle mass was reported to decrease approximately 3 to 8% per decade after age 30 years, with an even higher decrease after age 60 years [22]. The combination of muscle atrophy and poor muscle function is diagnosed as sarcopenia [23,24]. Thus, aging-related conditions predispose older individuals with T2DM to an increased risk for sarcopenia [25].

To explore the T2DM-SMA overlap, our present review (i) compiles evidence of this clinical concurrence and the causes and consequences of the T2DM-SMA association; (ii) reviews the pathophysiological and biochemical mechanisms underlying the development of SMA in people with T2DM and vice versa; and (iii) highlights the importance of treating muscle loss and impairment in T2DM-SMA through lifestyle interventions that include muscle-targeted medical nutrition therapy and physical activity. By better understanding the relationship between T2DM and SMA, we aim to advise optimal care strategies that can help prevent or delay the worsening of both conditions.

## 2. T2DM and SMA/Sarcopenia Risks Rise When These Threats Compound

The prevalence of SMA, or the more extreme condition of sarcopenia, is notably higher when diabetes is also present. An early study by Park et al. found that muscle mass decline was 2-fold faster in people with T2DM versus those without [3]. Results from an epidemiological study showed that the risk for sarcopenia was 1.5 to 2-fold greater in those with T2DM compared to those who were euglycemic [4,26,27].

Also, older adults with T2DM showed an accelerated decline in leg lean mass, muscle strength, and functional capacity when compared with normoglycemic control subjects [28]. Similarly, a study of older Japanese adults (≥65 years) showed sarcopenia in 12.2% of individuals without diabetes and 20.9% of those with T2DM [5]. Other researchers found that 30% to 50% of older patients (≥65 years) with T2DM experienced moderate-to-severe muscle loss; such muscle loss was 4 to 5 times more common in older people with diabetes compared to older populations in general [3,28,29].

Studies using tools to detect muscle atrophy and its response to treatment further support the association between T2DM and SMA. A recent cohort study and meta-analysis also showed inverse associations between muscle strength and T2DM; for each 5 kg higher handgrip strength, there was a 5% lower risk of T2DM, thus suggesting relative handgrip strength as a predictor of incident T2DM in older adults [30]. In yet another cross-sectional analysis of adults ≥ 20 years old (n = 13,644) subjects of the National Health and Nutrition Examination Survey (NHANES III) in the US, researchers found that for each 10% increase in skeletal muscle index, there was an associated 11% relative reduction in insulin resistance (IR) (assessed by the homeostasis model) [31].

## 3. Associations of T2DM and SMA Lead to Adverse Clinical Outcomes

Muscle loss and T2DM share common pathways, and both lead to impaired glucose homeostasis. Specifically, researchers have found notable commonalities in pathways associated with the onset of diabetes and SMA in adults [32]. A prospective, community-based study in older adults in China demonstrated that low baseline muscle mass and loss of muscle mass over time predicted T2DM incidence [32]. By contrast, effective correction of poor glycemic control in T2DM patients (shown by a decrease in glycated hemoglobin [HbA1c] by 1% or more) was significantly associated with increased skeletal muscle mass and faster gait speed [33].

Adverse clinical consequences of SMA in T2DM include impaired physical performance and decreased quality of life [12]. In addition, hyperglycemia in T2DM can lead to macrovascular and microvascular complications that aggravate skeletal muscle health [1,34], which can lead to increased mortality [35]. Taken together, this evidence demonstrates that preserving muscle health can have a major impact on diabetes management and the overall health of people with T2DM.

## 4. Skeletal Muscle as a Key Regulator of Glucose Homeostasis

Muscle is increasingly recognized as an active metabolic organ with a critical role in regulating postprandial glucose uptake for metabolism or storage [11]. Since skeletal muscle accounts for the removal of about 80% of the body’s glucose from circulation [36], the association of muscle loss with T2DM is not surprising [37]. Furthermore, T2DM is associated with IR, i.e., impaired insulin signaling at the cellular level, which represents a loss of muscle quality due to the lowered ability of muscle to transport and metabolize glucose. The relationship between IR and T2DM is recognized as a compelling reason to start screening adults for impaired glucose disposal early—beginning at age 35 years—using fasting plasma glucose tests, oral glucose tolerance tests, or HbA1c determinations to identify impaired glucose disposal [38,39,40]. Since skeletal muscle is recognized as an important regulator of glucose homeostasis, the maintenance or restoration of muscle has a pivotal role in diabetes management and progression.

## 5. The Vicious Cycle of Diabetes and Skeletal Muscle Atrophy

**Figure 1:**
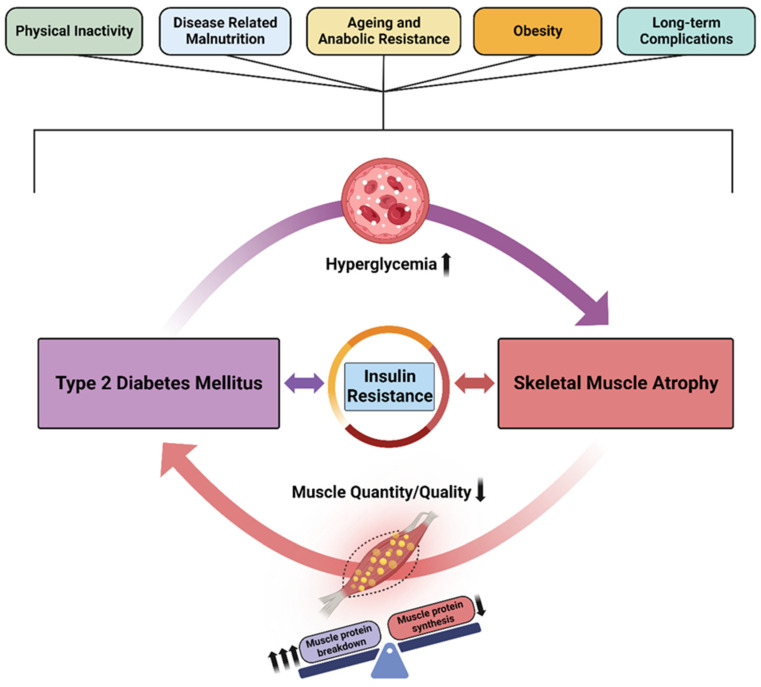
The vicious cycle of type 2 diabetes mellitus and skeletal muscle atrophy. Insulin resistance is the common and central element governing the association between T2DM and muscle atrophy. Low skeletal muscle mass and poor quality of skeletal muscle tissue result in impaired glucose uptake and increased blood glucose levels. Sustained elevation of blood glucose can lead to cardiovascular complications, which further impair blood circulation and glucose disposal.

Some researchers have described the relationship between T2DM and muscle loss as a vicious cycle [41,42,43]. T2DM increases the risk of and worsens muscle loss, and vice versa (Figure 1). Similarly, the back-and-forth effects of diabetes and sarcopenia have been described as bidirectional [34,44] or as a convergence of conditions [45].

Other factors—physical inactivity, disease-related malnutrition, anabolic resistance of aging, inflammation associated with obesity or other chronic health conditions, and low physical activity—can worsen the risk for both diabetes and sarcopenia, in turn leading to physical disability and other hyperglycemia-related health complications.

## 6. Biochemical Mechanisms Underlying Impaired Glucose Regulation and Muscle Dysfunction

**Figure 2:**
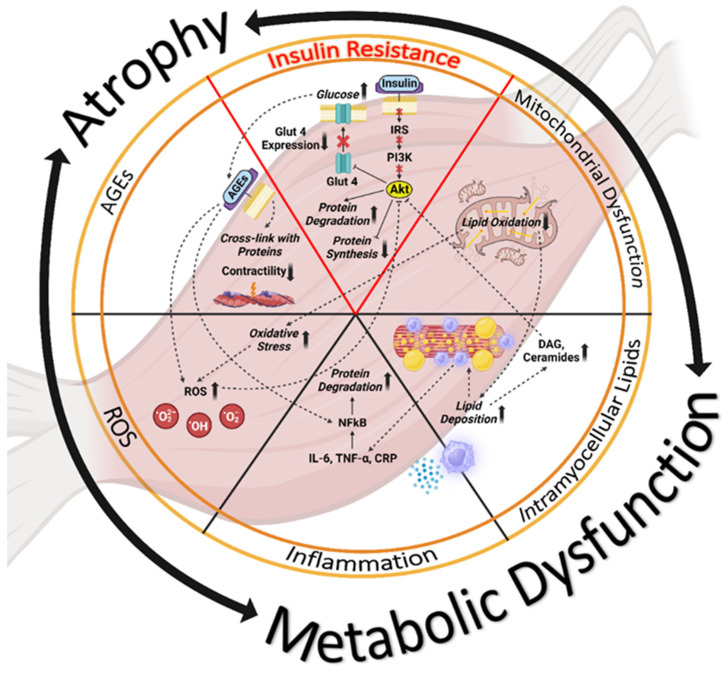
Biochemical mechanisms of impaired glucose uptake and metabolism in T2DM and its association with SMA. Alterations in the cellular and molecular mechanisms in a state of insulin resistance include inhibition of IRS-PI3K-AKT-mTOR signaling cascade, disruption of GLUT4 trafficking, low mitochondrial content, reduced ATP synthesis, excess oxidative stress, elevated production of lipid intermediates like DAG and ceramides as well as proinflammatory cytokines due to intramyocellular lipid deposition. In addition, the accumulation of AGEs induces changes in the myofibrillar protein structure. Abbreviations: IRS, Insulin Receptor Substrate; PI3K, Phosphoinositide 3-kinase; Akt, Ak strain transforming protein kinase; Glut 4, Glucose Transporter Type 4; AGEs, Advanced Glycation End products; ROS, Reactive Oxygen Species; DAG, Diacylglycerol. IL-6, Interleukin-6; TNF-α, Tumor Necrosis Factor-alpha; CRP, C-reactive protein; NFkB, nuclear factor kappa light chain enhancer of activated B cells.

IR and impaired glucose uptake in skeletal muscle are associated with a range of adverse conditions, including mitochondrial dysfunction, ectopic fat accumulation and increased intramyocellular lipid deposition, muscle inflammation with protein degradation in muscle tissue, oxidative stress with the formation of reactive oxygen species (ROS), and the formation of toxic advanced glycation end products (AGE) (Figure 2). Recognition of contributing mechanisms can lead to better understanding and improved treatment for T2DM and its associated conditions of SMA and sarcopenia [12]. Below, we briefly review cellular and molecular mechanisms that contribute to muscle dysfunction in people with T2DM and SMA:

Insulin resistance. Of the multiple causes of skeletal muscle impairment, IR plays a prominent role. IR is characterized by the blunting of the signaling pathways normally initiated by the binding of insulin to its cell surface receptor in target tissues. Insulin resistance leads to the downregulation of muscle contractile protein synthesis by inhibiting insulin’s anabolic IRS-PI3K-AKT-mTOR signaling pathway and simultaneously upregulating protein degradation via the ubiquitin–proteosome pathway. In addition, reduced skeletal muscle insulin signaling results in impaired translocation of GLUT4 glucose transporters to the myocyte cell surface for glucose uptake and use in skeletal muscle [46]. The resulting loss of muscle mass leads to both reduced glucose disposal and lowered mass and strength [37].

Mitochondrial dysfunction. Mitochondrial dysfunction underlies IR and muscle atrophy [47]. Mitochondria are intracellular organelles that generate adenosine triphosphate (ATP) for the chemical energy needed to power cellular functions and to produce ROS. With an overload of glucose, mitochondria produce more ROS and more oxidative stress, ultimately decreasing ATP production [48]. Age- and diabetes-related muscle loss has been associated with mitochondrial abnormalities with respect to content, size, and morphology as well as function. Compared to younger adults, those who are older exhibit lower oxidative capacity per unit muscle, which results in mitochondrial impairment [49,50]. Newer research results indicate that the accumulation of mitochondrial DNA (mtDNA) mutations and mtDNA copy number depletion may contribute to mitochondrial dysfunction in T2DM [51].

Intramyocellular fat deposition. When IR occurs in adipose tissue, excess fat is released into the bloodstream, and lipids eventually accumulate in other organs (skeletal muscles and liver) that are not equipped to store fat [52]. Increased intracellular lipid accumulation leads to the buildup of detrimental lipid intermediates such as diacylglycerols and ceramides, which further worsen muscle quality and lessen muscle quantity [53].

Intramuscular inflammation. Lipid-caused intramuscular inflammation is another possible mechanism underlying the link between diabetes and muscle atrophy. The increased infiltration of proinflammatory molecules such as interleukin 6 (IL-6), tumor necrosis factor-α (TNF-α), and C-reactive protein (CRP) leads to muscle inflammation with resultant loss of insulin sensitivity and muscle atrophy by way of protein degradation mediated by nuclear factor kappa light chain enhancer of activated B cells (NF-κB) and other signaling pathways [54].

Reactive oxygen species and oxidative stress. Oxidative stress is a general condition that results from reduced antioxidant capacity associated with aging and from increased production of reactive oxygen species (ROS) in diabetes. Such stress impairs insulin signaling and disturbs glucose uptake in skeletal muscle, thus contributing to hyperglycemia [2]. ROS activate the degradation of proteins with a parallel inhibition of protein synthesis, leading to progressive deterioration of muscle mass and muscle metabolic functions [55,56].

Advanced glycation end products (AGEs). AGEs are a complex and chemically diverse group of molecules produced from the non-enzymatic reaction between carbonyl groups of reducing sugars and free amino groups of proteins, lipids, and nucleic acids, followed by further rearrangement reactions yielding irreversible end-products. AGEs are generated inside the organism as part of normal metabolism or provided exogenously by increased consumption of highly processed food (via the Maillard reaction) as well as cigarette smoking. AGEs have been implicated in many physiological and pathological signaling pathways associated with aging, IR, and hyperglycemia [57]. The pathologic effects of AGEs in skeletal muscle are related to their ability to increase cross-linking of proteins, altering their structure and function and thereby interfering with contractility. Additionally, AGEs can promote oxidative stress, inflammation, and mitochondrial dysfunction by binding with cell surface receptors [58].

## 7. Overall Care Strategies for T2DM and SMA

Malnutrition and low muscle mass are prevalent conditions among adults with diabetes or other chronic diseases [59,60]. Clinical care strategies for treating people with T2DM and SMA include (i) medications to improve glycemic control, (ii) dietary guidance and additional support with diabetes-specific oral nutritional supplements (ONS), and (iii) physical activity [61].

### 7.1. Diabetes-Focused Nutritional Guidance

All individuals with T2DM can benefit from nutritional guidance to promote healthy eating patterns, achieve and maintain a healthy body weight, and delay or prevent diabetes complications. For people with diabetes, regular consultations with dietitians or other nutrition experts are key to optimal outcomes [60].

Here, we focus on preventive and restorative nutritional support for muscle health, especially nutritional interventions that enhance muscle anabolism and reduce catabolism, thus helping maintain physical function [18,58,62]. Along with consuming healthy proportions of macronutrients (protein, carbohydrates, and fats), specific functional nutrients (vitamin D, specific amino acids, HMB, omega-3 fatty acids, and polyphenols) appear to lessen inflammatory processes and reduce oxidative stress (Table 1).

Adequate and balanced intake of macronutrients (protein, carbohydrate, and fat) is important to achieve metabolic control in T2DM and to sustain muscle mass and functionality. Adequate protein supports muscle synthesis, while sufficient energy (as fat and carbohydrate) is important to prevent the metabolic breakdown of skeletal muscle when its protein is used as an alternative source of energy [11,63].

While standard recommendations for protein intake are 0.8 g/kg body weight, adequate intake for adults who are older and experiencing chronic diseases such as T2DM may be increased by 20% or more [62,64,65]. In fact, protein intake (g/kg BW) was positively associated with skeletal muscle index (r = 0.262, *p* = 0.010 in men and r = 0.295, *p* = 0.013 in women) in a cross-sectional study including elderly outpatients with T2DM (at high risk of sarcopenia) [66]. Several studies have focused on the effect of protein combined with regular exercise, and a recent publication investigated the effect of exercise with or without adequate protein intake on muscle mass in 214 elderly patients with T2DM. This study found that, compared with patients with exercise and adequate protein intake (≥1.2 g/kg ideal BW/day), those with exercise and inadequate protein intake have up to a 4-fold higher risk of decreased skeletal muscle index [67].

Individual amino acids may also play a role in glucose homeostasis in the body. The conditionally essential amino acid L-arginine may improve insulin sensitivity and glucose metabolism [68], although mechanisms are not yet fully understood [69]. A suggested mechanism underlying IR is related to the deficient production of nitric oxide (NO), a nitrogen-free radical produced continuously from L-arginine [70]. Among people with reduced oral glucose tolerance and metabolic syndrome, supplementation of L-arginine (~6 g orally/day) for 18 months led to a significant restoration of normal glucose tolerance [71,72]. A comprehensive review of the effects of L-arginine in diabetes, including human and animal studies, has been recently published [73]. The authors concluded that L-arginine supplementation could be a promising strategy, especially for those patients in the early stages of diabetes, mainly to slow down complications. However, despite all this evidence, more randomized and long-term clinical trials are warranted to assess the beneficial effects of L-arginine on blood glucose management during diabetes progression.

Supplementation with the amino acid L-lysine has been suggested as a promising ingredient to improve glycemic control, decrease protein glycation, and lower IR in T2DM patients [74,75]. In a single-evaluator-blinded, randomized clinical trial in T2DM patients, using anti-diabetes medication, L-lysine supplementation (orally administered with 3 g/day for 3 months) was able to inhibit protein glycation, improve glycemic control, and increase antioxidant markers [74]. It seems L-lysine may act as an inhibitor of the non-enzymatic glycation of some proteins and may protect protein folding in both in vitro experiments and animal models of diabetes [76]. Although clinical evidence behind the L-lysine role in diabetes is still scarce, current evidence supports that L-lysine supplementation may also act as an effective complementary treatment for glucose management.

L-leucine, an essential branched-chain amino acid, has been demonstrated to be a potent modulator of protein turnover, particularly protein anabolism [77,78,79]. Beta-hydroxy beta-methylbutyrate (HMB) is an active metabolite of leucine [80]. Apart from being an important anabolic stimulus, HMB is the most clinically studied leucine metabolite to prevent muscle protein breakdown, especially under hypercatabolic conditions. The research team of Lin et al. conducted a meta-analysis of nine randomized, controlled trials to evaluate the effect of the nutritional ingredient HMB on the muscle strength of older adults. They found that dietary supplementation of HMB and consumption of preparations containing HMB (providing 1.5–3 g/day) contribute significantly to increased muscle strength in older people [81]. Further, a recent review article found that HMB had a positive effect on body composition and strength, especially in bedridden or sedentary older people, because of its anticatabolic properties [82].

The nutritional combination of HMB, arginine, and lysine showed benefits for T2DM and SMA—both in animals and humans. In rats, the combination of HMB, lysine, and arginine was effective in controlling blood glucose levels as well as in preventing skeletal muscle atrophy associated with the progression of diabetes [83]. Results of a human study of targeted nutrition in elderly women showed that daily supplementation with a combination of HMB, arginine, and lysine for 12 weeks positively altered measurements of functionality, strength, fat-free mass, and protein synthesis, thus indicating that this nutritional strategy can improve muscle health in elderly women [84].

Carbohydrates are the body’s primary source of energy and the brain’s preferred energy source. Intake of simple sugars like glucose and fructose must be limited, as they worsen metabolic complications in skeletal muscle, adipose tissue, and the liver and can lead to adverse clinical outcomes [85]. However, population studies suggest that the total amount of carbohydrates is less important than the carbohydrate type for the risk of chronic diseases such as T2DM [86].

Slowly digested carbohydrates (SDC) avert postprandial glucose spikes; natural foods with SDC include minimally processed grains, legumes, and whole fruits [86]. Isomaltulose is a disaccharide source of glucose and fructose linked by a different glycosidic bond (1,6). Like sucrose, it is fully available to the body but slowly released, thus resulting in a much slower, lower, and longer-lasting blood glucose response compared to sucrose [87]. Isomaltulose may provide glucose in a more balanced and sustained way [87,88].

A systematic review demonstrated that after consuming a drink with isomaltulose, a lower elevation of blood glucose was observed in subjects with diabetes compared to a drink including sucrose [89].

In addition, sucromalt contains the same amount of carbohydrates as high fructose corn syrup but causes lower glucose and insulin responses [90]. These effects are beneficial to people with diabetes because they help manage their blood glucose levels. As a low-glycemic alternative to other types of carbohydrates, sucromalt provides desirable sweetness to foods while taking significantly longer for the body to digest and absorb [90].

Dietary fibers are complex carbohydrates that human digestive enzymes are not able to break down. Instead, these polysaccharides are metabolized by gut microbes, which break down the fibers enzymatically to generate short-chain fatty acids important as energy sources to maintain cells in the gut epithelium [91].

Myo-inositol, a sugar alcohol carbohydrate, mediates signal transduction in response to insulin and several other growth factors; as such, it appears to play a role in the reduction of IR [92,93], in turn improving blood glucose and HbA1c levels [94]. A recent systematic review compiled the current scientific evidence reporting the benefits of myo-inositol on diabetes-related parameters [95]. It included 16 studies on diabetes using different doses and durations and examined the relationship between diabetes and inositol supplementation. For example, a 3-month study of myo-inositol (550 mg) and its isomer d-chiro-inositol (138 mg) twice a day for treatment of people with T2DM yielded significantly decreased fasting blood glucose levels (192.6 ± 60.2 decreased to 160.9 ± 36.4; *p* = 0.02) and HbA1c levels (8.6 ± 0.9 decreased to 7.7 8 ± 0.9; *p* = 0.02 [96]. Researchers also found that daily myo-inositol supplementation significantly decreased the incidence of gestational diabetes mellitus [97].

Another systematic review included 20 randomized controlled trials with a total of 1239 subjects and reported that myo-inositol supplementation was associated with improved insulin sensitivity, independent of weight loss [98]. One study conducted in 66 patients with T2DM received inositol supplementation or placebo for 12 weeks [99]. The inositol group improved mean HbA1c levels (lowered by 0.65 vs. 0.01), lowered fasting plasma glucose by 52.58 vs. 2.21, and lowered IR (HOMA-IR by 6.08 vs. 0.71. This study concluded that myo-inositol supplementation might support better blood sugar management in T2DM. Therefore, current literature demonstrates that myo-inositol may be effective in the treatment of diabetes.

Diets rich in monounsaturated fatty acids (MUFA) and polyunsaturated fatty acids (PUFA) and low in saturated fatty acids (SFA), trans fat, and cholesterol are associated with reduced cardiovascular disease (CVD) risk factors [100].

A 2020 systematic review and meta-analysis by Sanz-Paris et al. compared a diabetes-specific formula (DSF) high in MUFA vs. a standard diabetes formula (STDF) in adult patients with diabetes or stress-induced hyperglycemia. A total of 18 RCTs were included, of which 845 adults met the inclusion criteria. The meta-analysis showed a significantly reduced postprandial glucose peak and plasma insulin in patients with diabetes consuming DSF high in MUFA vs. those consuming STDF and demonstrated a reduction in glycemic variability (improved glycemic control), reduced HbA1c levels (improved long-term glycemic control), lower insulin doses (reduced use of medication), and improved lipid metabolism (less cardiovascular risk) [101].

Consumption of PUFA is also protective, while high total fat and saturated fatty acid intake are recognized as detrimental to skeletal muscle [20]. Specifically, dietary intake of anti-inflammatory fatty acids can help offset the risk for advanced complications of diabetes. For instance, omega-3 fatty acids can resolve inflammation [102,103], while dietary omega-6 fatty acids can contribute to worsened inflammation and a higher risk for cardiovascular complications [104]. However, a recent meta-analysis observed a significant inverse association with T2DM risk across categories of α-linolenic acid (ALA) (relative risk [RR]: 0.89, 95% confidence interval [CI]: 0.82–0.96) and eicosapentaenoic acid (EPA) (RR: 0.85, 95% CI: 0.72–0.99) biomarkers, suggesting that dietary recommendations may include omega-3 to help maintain an overall lower risk of developing T2DM [105]. Based on findings from a systematic review and meta-analysis, researchers concluded that increased dietary intake of omega-3 fatty acids may be beneficial for the prevention of T2DM [106].

On the other hand, EPA, docosahexaenoic acid (DHA), and ALA supplementation with or without exercise have been reported to improve muscle strength and functionality in older adults within a dose range of 2–3 g/day of omega-3 (EPA and DHA), suggesting a beneficial effect of an EPA:DHA ratio of 2:1 [107,108].

Vitamin D plays many important roles in the body, including bone health, reduction in inflammation, modulation of such processes as cell growth, neuromuscular and immune function, and glucose metabolism [109,110,111,112,113,114,115]. Age, mobility, and concurrent disease can increase the risk of vitamin D deficiency, and in particular, older adults are at increased risk of poor vitamin D status due to reduced exposure to sunlight, compromised endogenous synthesis and renal conversion to the active form of the vitamin, and reduced dietary intake [116,117]. Vitamin D deficiency has been shown to be as high as 58% in medical in-patients with disease-related malnutrition, and deficiency is associated with negative long-term health outcomes [110].

Vitamin D supplementation has been shown to increase serum 25(OH)D and reduce IR [118]. Deficient vitamin D status is associated with higher HbA1c and complications in people with diabetes, while vitamin D supplementation is associated with improvements in glycemia and inflammation. The meta-analysis by Mousa et al. demonstrated that vitamin D supplementation can improve the state of low-grade inflammation in T2DM [119]. In addition, Wu et al. showed that vitamin D supplementation was associated with improved glycemic control, measured by HbA1c and fasting blood glucose levels, in patients with T2DM and vitamin D deficiency [120]. Therefore, preventing deficiency of vitamin D may be helpful in improving glucose homeostasis.

Like vitamin D, many dietary natural products belonging to Mediterranean foods, such as polyphenols, have been proposed as effective therapeutic agents for managing and preventing T2DM, either independently or in combination with other anti-diabetic drugs [121,122]. These bioactive molecules exhibit anti-diabetic properties through several mechanisms, including protection of β-cell function, inhibition of carbohydrate digestion and glucose absorption, improvement of insulin action, modulation of the intestinal microbiota, optimization of adipose tissue metabolism, and inhibition of AGE formation [123].

**Table 1:** Interventions for type 2 diabetes mellitus and skeletal muscle atrophy: medical nutrition with functional ingredients.

Medical Nutrition Therapy (MNT)		Scientific Evidence
**Protein** 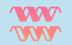	It is necessary for muscle turnover, malnourished patients require high protein intake (1.2–1.5 g/kg body) [11,61,62,63,64,65,66,67].	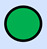
**L-Arginine** 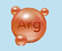	Promotes vasodilation, blood flow and it can counteract insulin resistance [68,69,70,71,72,73].	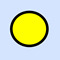
**L-Lysine** 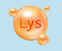	It can inhibit nonenzymatic glycation of some proteins and conserve protein folding [74,75,76].	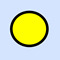
**L-Leucine** 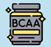	An essential amino acid and structural component of most proteins that stimulates muscle protein synthesis [77,78,79].	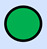
**HMB** 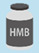	Muscle-targeted metabolite that inhibits muscle protein breakdown and supports muscle function [80,81,82,83,84].	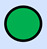
**Low GI CHO** 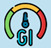	Slow-digested CHO effectively reduce postprandial glucose and insulin responses [86,87,88,89,90].	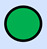
**Dietary Fiber** 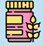	Fibers are important for the control of postprandial glycemic response and can have prebiotic effects [91].	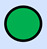
**Myo-inositol** 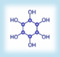	It can help improve blood glucose management (measured by HbA1c) [92,93,94,95,96,97,98,99].	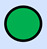
**MUFAs** 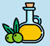	MUFAs are important to support better glycemic control mainly by reducing glycemic variability and A1C levels [101].	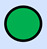
**Omega-3 FAs** 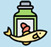	Actively participate in inflammation resolution. Associated with benefits on fasting blood glucose, insulin resistance and decreased risk of T2DM [102,103,104,105,106,107,108].	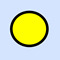
**Vitamin D** 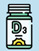	Diabetic patients have lower serum Vitamin D. This deficiency can affect insulin sensitivity and secretion [109,110,111,112,113,114,115,116,117,118,119,120].	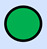
**(Poly)phenols** 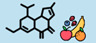	Modulate oxidative stress and anti-inflammatory responses and can contribute to counteract hyperglycemia in T2DM individuals [121,122,123,124,125,126,127,128].	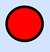
**Creatine** 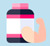	Its combination with physical exercise can reduce A1C levels and improve muscle strength and function [129].	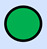

Scientific Evidence: color-based score according to available scientific evidence reporting a positive effect of the ingredient on muscle health and/or glycemic response. Abbreviations: low GI CHO, low glycemic index carbohydrates; MUFAS, monounsaturated fatty acids; FAs, fatty acids.

The beneficial role of polyphenols in alleviating muscle atrophy and improving skeletal muscle regeneration, partly due to their antioxidant, anti-inflammatory, and immunomodulatory activities, has been extensively investigated in a wide range of in vitro and in vivo experiments [124,125]. In addition, several pilot studies in diabetes subjects have demonstrated that the administration of polyphenols improves skeletal muscle mitochondrial function, reduces oxidative stress, and improves myofiber regeneration [126,127,128]. However, more investigation is needed to elucidate how diabetes influences the benefits of dietary polyphenols, their bioavailability, and bioactivity, as well as the duration of supplementation and the mechanism involved in optimizing the clinical use of these natural products in the management of muscle atrophy in people with diabetes.

Creatine is a supplemental nutrient that is widely used to improve power, strength, muscle mass, and performance. In combination with exercise training, creatine may improve glucose metabolism in healthy individuals and insulin-resistant individuals, as with T2DM [129]. The suggested mechanism is increased glucose transport into muscle cells as a result of enhanced translocation of type 4 glucose transporters (GLUT-4) from the muscle cell interior to the cell surface, an insulin-stimulated response [129].

### 7.2. Physical Activity and Muscle-Strengthening Exercises

Physical activity continues to be an important way to support glucose disposal in skeletal muscle, to preserve or improve muscle health, and to counteract insulin resistance [27,37]. Physical activity is thus particularly important for people with diabetes or at risk of diabetes due to sarcopenic obesity [67,130,131].

Muscle-strengthening exercises, along with regular physical activity, can improve both skeletal muscle quantity and quality [27]. Resistance exercises promote muscle protein synthesis for increased muscle mass and decrease adiposity for reduced lipid infiltration into the muscle [61].

Several exercise schemes have been proposed to improve glucose disposal in individuals with T2DM. The study by Hansen et al. [132] explored whether exercise training in the fasted state may be relevant to patients with T2DM or IR. This study found that long-term exercise training in the fasted state in healthy subjects was associated with improved insulin sensitivity, basal muscle fat uptake capacity, and oxidation, which may be promising to improve insulin sensitivity or glycemic control in patients with T2DM, although more research is needed.

In addition, a recent publication described how specific training schemes such as strength-based training can be an important and valid methodology to reduce sarcopenia-related problems, which may be useful in T2DM-associated SMA. The authors proposed weight resistance training as part of an integral strategy to counteract sarcopenia [133].

In particular, a program of 8 weeks of suspension-based resistance training (S-RT) has shown benefits on glycemic and lipid profiles in people with T2DM [134].

In another study [135], the effect of high-intensity interval training (HIIT) on glycemic control, physical fitness, and body composition in individuals with T2DM was also assessed. Exercise training increased the VO2 peak more in the HIIT group (20% ± 20%) compared with the endurance training group (8% ± 9%). Moreover, visceral fat mass, HbA1c, fasting glucose, postprandial glucose, glycaemic variability, and HOMA-IR decreased after HIIT, suggesting that HIIT may be an important strategy for individuals with T2DM [136].

Altogether, current research advises on the benefits different training schemes can provide to individuals with diabetes to improve glucose management as well as muscle health as a potential strategy to counteract SMA.

## 8. Summary and Recommendations for Future Research

T2DM and SMA are common and interrelated clinical conditions today. In adulthood, the aging process is associated with an increasing likelihood of experiencing T2DM and SMA (Box 1). These conditions are associated with the loss of skeletal muscle, which may ultimately lead to poor mobility, frailty, loss of independence, a lower quality of life, and even an increased risk of mortality [5,130]. Thus, healthy muscle becomes a key target for healthy aging in general. For people who are aging well, maintaining muscle is a general target [61,130]. With further advancing age and declining wellness, care shifts toward preventing frailty and disability by restoring lost muscle mass, strength, and function. In terms of clinical care for adults with T2DM and SMA risk, early care focuses on treating prediabetes and sarcopenia risk with strategies that build and maintain muscle, while later care focuses on achieving and sustaining glucose control and restoring lost muscle [61,130].

Box 1 **Type 2 diabetes and skeletal muscle atrophy.**

- **T2DM and SMA are increasingly common and interrelated clinical conditions.**
- **Both T2DM and SMA are worsened by aging, poor nutrition (under- or over-nutrition), acute and chronic diseases, and limited physical activity (lifestyle- or disability-related).**
- **Each condition worsens the consequences of the other by affecting overlapping pathophysiological mechanisms.**
- **Impaired muscle health can in turn lead to adverse effects on a person’s mobility and physical function, on quality of life, and on survival.**
- **Nutritional interventions that include functional ingredients targeting blood glucose management and muscle health are key to the clinical management of patients with T2DM and SMA.**
- **Individualized prescriptions for physical activity are also key to achieving optimal health outcomes.**

To target both muscle loss and T2DM progression, we advise care that includes a combination of nutritional and exercise strategies. Specialized nutrition, including functional ingredients such as slowly digested and low-glycemic index carbohydrates, high protein, anabolic amino acids, and HMB, can be particularly beneficial.

## 9. Conclusions and Future Directions

Today, T2DM and SMA are increasingly common conditions that are linked in a bidirectional relationship by the condition of IR. The use of key functional ingredients that target muscle health and blood glucose management, along with individualized guidance for physical activity, are key to maintaining and restoring muscle health. Individualized prescriptions for medications are also essential to achieve optimal glucose regulation and to maintain and restore skeletal muscle.

Although several ingredients have been proposed as good candidates to address SMA while providing good glycemic control, such as proteins, low GI carbohydrates (SDC and dietary fiber), amino acids, HMB, polyphenols, myo-inositol, omega-3, vitamin D, and creatine, more randomized and placebo-controlled studies are warranted to demonstrate the optimal doses that are associated with skeletal muscle health benefits at longer treatment durations in a larger sample size of individuals with T2DM. Current evidence suggests that optimal nutrition in combination with resistance training seems to be the best strategy for these individuals, but more clinical trials elucidating the role of specific nutrition interventions together with targeted training schemes are still needed.

In conclusion, better maintenance of muscle health holds huge potential for improving general health and quality of life for adults with T2DM. Further, extending the interval that people with T2DM live independently and in good health is expected to help lower costs for hospitalizations and for other supportive care such as home health services and assisted living facilities.

## Data Availability

Not applicable.
